# Feeding a High-Concentrate Corn Straw Diet Induced Epigenetic Alterations in the Mammary Tissue of Dairy Cows

**DOI:** 10.1371/journal.pone.0107659

**Published:** 2014-09-15

**Authors:** Guozhong Dong, Min Qiu, Changjin Ao, Jun Zhou, Khas-Erdene, Xi Wang, Zhu Zhang, You Yang

**Affiliations:** 1 College of Animal Science and Technology, and Key Laboratory of Grass and Herbivores of Chongqing, Southwest University, Beibei, Chongqing, China; 2 College of Animal Science, Inner Mongolia Agricultural University, Hohhot, Inner Mongolia, China; Monash University, Australia

## Abstract

**Purpose:**

The objective of this study was to investigate the effects of feeding a high-concentrate corn straw (HCS) diet (65% concentrate+35% corn straw) on the epigenetic changes in the mammary tissue of dairy cows in comparison with a low-concentrate corn straw (LCS) diet (46% concentrate+54% corn straw) and with a low-concentrate mixed forage (LMF) diet (46% concentrate+54% mixed forage).

**Experimental Design:**

Multiparous mid-lactation Chinese Holstein cows were fed one of these three diets for 6 weeks, at which time blood samples and mammary tissue samples were collected. Mammary arterial and venous blood samples were analyzed for lipopolysaccharide (LPS) concentrations while mammary tissue samples were assayed for histone H3 acetylation and the methylation of specific genes associated with fat and protein synthesis.

**Results:**

Extraction of histones and quantification of histone H3 acetylation revealed that acetylation was significantly reduced in cows fed the HCS diet, as compared with cows fed the LCS diet. Cows fed the HCS diet had significantly higher LPS concentrations in the mammary arterial blood, as compared with cows fed the LCS diet. We found that the extent of histone H3 acetylation was negatively correlated with LPS concentrations. The methylation of the stearoyl-coenzyme A desaturase gene associated with milk fat synthesis was increased in cows fed the HCS diet. By contrast, methylation of the gene encoding the signal transducer and activator of transcription 5A was reduced in cows fed the HCS diet, suggesting that feeding a high-concentrate corn straw diet may alter the methylation of specific genes involved in fat and protein synthesis in the mammary tissue of dairy cows.

**Conclusions:**

Feeding the high-concentrate diet induced epigenetic changes in the mammary tissues of dairy cows, possibly through effecting the release of differing amounts of LPS into the mammary blood.

## Introduction

Epigenetics is defined as the study of heritable changes in gene expression that are independent of DNA sequence [1]. Major epigenetic events include acetylation and methylation of histones, DNA methylation, and gene regulation by non-coding RNAs. Histone binding to DNA in the eukaryotic nucleus modifies the properties of DNA. Various histone modifications include acetylation, methylation, phosphorylation, and ubiquitination [2]. The acetylation and methylation of histones H3 and H4, which occur at lysine or arginine residues, are the most common and well-studied histone modifications. DNA methylation refers to the addition of a methyl group at cytosine residues of the DNA template, which occurs predominantly in cytosine-phosphate-guanine (CpG) dinucleotides in mammals [3]. Epigenetic modifications of histones and DNA occur in response to environmental factors. Diet, toxins, pollutants, disease and other environmental factors may have profound impacts on gene expression through epigenetic regulation. The potential of epigenetic regulation to impact mammary function in dairy cows has received attention in recent years [4] [5].

In dairy production systems, cattle are often fed high-concentrate diets due to a seasonal or regional lack of quality forage material. The feeding of dairy cows diets that contain high proportions of concentrate to support high milk production has been associated with a high incidence of subacute ruminal acidosis (SARA) [6] [7] [8]. SARA is a digestive disorder that poses a health threat to lactating dairy cows and has become the most important metabolic disease in dairy production in the world. The high rumen digestibility of most grains in concentrate mixtures increases the production of volatile fatty acids in the rumen and causes a corresponding drop in rumen pH [6] [9] [10], which can result in alterations in the rumen environment, leading to changes in the composition of rumen microbiota [11] [12] and the accumulation of endotoxin (lipopolysaccharide; LPS), a potentially harmful cell-wall component of gram-negative bacteria [7] [13] [14]. LPS can translocate into the bloodstream across the epithelial barrier of the gastrointestinal tract [15] [16] [17], triggering inflammatory responses in cows [15] [18] [19].

LPS may also induce harmful epigenetic changes in the mammary tissue of dairy cows. However, the association between LPS and epigenetic changes in the mammary tissue of dairy cows during the feeding of diets high in concentrate has not yet been documented. In addition, epigenetic controls of milk gene expression through histone modification and DNA methylation during the feeding of high-concentrate diets to dairy cows are unknown. In many countries, corn straw is frequently used in the diets of cows due to a lack of quality roughage, requiring an increase in the proportion of concentrate in the diet to meet the nutritional requirements for lactation. We hypothesized that increasing the concentrate proportion in a corn straw-based diet will result in increased entry of endotoxin into the mammary gland and thus elicit epigenetic alterations in the udder, which may exert an adverse effect on milk gene expression. Therefore, the objective of this study was to evaluate the effects of feeding a high-concentrate corn straw (HCS) diet on the epigenetic changes in the mammary tissue of dairy cows in comparison with a low-concentrate corn straw (LCS) diet and a low-concentrate mixed quality forage (LMF) diet.

## Materials and Methods

### Ethics statement

The study was approved by Animal Ethics Committee of Southwest University, and all experimental procedures and the care of the animals were in strict accordance with the “Guidelines on Ethical Treatment of Experimental Animals (2006, No. 398)” issued by the Ministry of Science and Technology of China. Blood and mammary tissue sampling described in detail below was performed under either general or regional anesthesia to minimize suffering and pain in the animals.

### Animals, diets and experimental procedure

Thirty second-parity Chinese Holstein cows in mid-lactation, averaging 543±57 kg of body weight and producing 24.32±3.86 kg milk per day at the onset of the experiment, were randomly assigned to 1 of 3 diets (n = 10 per treatment; Table 1). These diets included: 1) the LMF diet with a concentrate to roughage ratio of 46∶54, containing Chinese wildrye (*Aneurolepidium chinense*), alfalfa hay, and corn silage. This diet is commonly regarded as an excellent diet for lactating cows and served as a control in the study. 2) the HCS diet with a concentrate to roughage ratio of 65∶35, containing corn straw as the only roughage but otherwise identical (except fiber content) to the LMF diet. 3) the LCS diet with the same concentrate to roughage ratio (46∶54) as the LMF diet, and the LCS diet also contained corn straw as the only roughage material. Diets (the LMF and the HCS diets) were formulated based on the nutrient requirements of dairy cows, as recommended by NRC [20]. The LCS diet was formulated by simply replacing the forages of the LMF diet with corn straw. Diets were mixed and offered as total mixed ration twice daily (0830 and 1730 h). Orts were discarded before the next feeding each day and the amount of feed was adjusted to ensure a 5% feed residual. Cows were milked twice daily at 0800 and 1900 h. The experiment lasted 6 weeks. Cows were housed with free access to water. Cows in the experiment were observed daily for feed ingestion, and the rectal temperature and respiratory rates were also examined each day. The cows showed no clinical signs of infectious disease throughout the entire experimental period. This experiment was conducted at the Inner Mongolia Dairy United Technology Co., Ltd., Hohhot, China.

**Table 1:** Diet compositions.

	Diet^1^		
Item	LMF	HCS	LCS
Ingredient (% dry matter)			
Alfalfa hay	23.4	0.0	0.0
Corn silage	26.7	0.0	0.0
Chinese wildrye	3.7	0.0	0.0
Corn straw	0.0	35.0	53.8
Corn	24.6	35.3	24.6
Soybean meal	14.8	20.8	14.8
Whole cottonseed	5.1	7.2	5.1
Dicalcium phosphate	0.6	0.8	0.6
Salt	0.5	0.5	0.5
Premix^2^	0.6	0.6	0.6
Concentrate∶Roughage	46∶54	65∶35	46∶54
Nutrient composition (%) of dry matter)			
Net Energy (Mcal/kg)	1.50	1.54	1.40
Crude protein	16.8	16.9	13.9
Neutral detergent fiber	37.6	34.4	46.0
Acid detergent fiber	23.9	19.9	26.7
Non-fiber carbohydrate^3^	37.0	41.3	32.6
Ether extract	3.4	3.2	2.5
Calcium	0.5	0.5	0.4
Phosphorus	0.4	0.4	0.4

1 LMF, low-concentrate mixed forage diet; HCS, high-concentrate corn straw diet; LCS, low-concentrate corn straw diet.

2 Premix contains: 2,142 mg/kg Cu (as sulfate); 15,428 mg/kg Mn (as sulfate); 15,428 mg/kg Zn (as sulfate); 28 mg/kg Co (as chloride); 231 mg/kg I (as iodate); 57 mg/kg Se (as selenite); 2,285,000 IU/kg vitamin A; 457,000 IU/kg vitamin D; and 11,400 mg/kg vitamin E.

3 Non-fiber carbohydrate = 100−(% Neutral detergent fiber+% Crude protein+% Ether extract+% Ash).

### Blood sampling and analysis

Blood samples were obtained shortly before the morning feeding from the mammary (external pudendal) artery and the mammary vein, respectively, on day 6 and 7 of the last week of the animal trial. Cows were restrained in a standing posture during blood sampling. To keep cows sedated, 20 mL procaine hydrochloride was injected into the muscles near the artery blood collection point, located between the femoral and hip joints. The operator put one hand into the cow's rectum against the external pudendal artery and used the other hand to hold a 18#, 20 cm long sterile needle which was vertically penetrated into the fossa formed through convergence of the internal abdominal oblique muscle, the vastus lateralis muscle and the gluteus medius muscle, after shearing and disinfection. Blood samples were collected in 10-mL vacuum tubes containing heparin sodium anticoagulant and were stored on ice and centrifuged (Feige TDL-40B, Shanghai Anting Scientific Instrument Factory) within 10 min at 3,000× *g* and 4°C to harvest plasma. The plasma was immediately stored at −20°C until being analyzed for LPS concentrations. The concentrations of LPS in plasma were determined by chromogenic end-point assay using the limulus amebocyte lysate (LAL) test reagent kit (QCL-1000, Lonza Group Ltd., Basel, Switzerland) with a minimum detection limit of 0.1 EU/mL. Samples were initially treated as described by Khafipour et al. [15] to inactivate inhibitory factors in plasma. Then a metallo-modified polyanionic dispersant named Pyrosperse (Lonza Group Ltd.) was added to the samples at a ratio of 1/200 (vol/vol) before LAL testing. The LAL assay was performed by dispensing 50 µL of sample into a pyrogen-free glass tube in a 37°C water bath and mixing with 50 µL LAL reagent. After 10 min, 100 µL substrate solution prewarmed to 37°C was added. After 16 min, stop reagent and azoic reagent (Xiamen Limulus Experiment Factory, Xiamen, China) were added. Optical density at 545 nm was measured using a microplate reader (Synergy H4, BioTek, Winooski, VT).

### Mammary tissue sampling and analysis

Mammary tissue samples were obtained by a punch biopsy according to Baumgard et al. [21] with modifications. Cows were restrained from movement under general anesthesia. The mammary gland was exposed and 20 mL procaine hydrochloride was administered in a circular pattern surrounding the incision site. A 5–6 cm incision in the skin on the midpoint of the rear quarter of the right mammary gland was made after shearing and disinfection. Connective tissue was dissected away to reveal the gland capsule and mammary tissue biopsy (∼500 mg) was obtained and divided in half. One half of the sample was used in histone H3 acetylation assays while the other was used in DNA methylation assays. All samples were immediately stored in liquid nitrogen. After mammary tissue sampling was performed, the gland capsule, connective tissue and skin were sutured, and the animals were given intramuscular penicillin to prevent infections.

Total histones were extracted using the EpiQuik™ Total Histone Extraction Kit (Epigentek Group Inc.) according to the manufacturer's protocols. Histone H3 acetylation was quantified using the EpiSeeker Histone H3 Acetylation Assay Kit (Abcam). Histone H3 acetylation sites were identified by resolving histone H3 using sodium dodecyl sulfate-polyacrylamide gel electrophoresis and staining with Coomassie brilliant blue. The histone H3 band was excised and destained with 50% acetonitrile+25 mM ammonium bicarbonate. Histone H3 was digested with trypsin and peptides were analyzed using liquid chromatography-mass spectrometry (MS)×MS (Thermo Scientific Inc.). Peptide mass spectra were measured and analyzed to determine acetylation sites using Mascot software, with special attention paid to the detection of ions with a mass-to-charge ratio (m/z) of 126.1, which is diagnostic for lysine acetylation [22]. Lysine-acetylated peptides were also confirmed by detecting mass differences of 170 Da between adjacent b-ions or y-ions.

Total DNA from the mammary tissue was extracted using a reagent kit (Tiangen, Beijing, China) and used in DNA methylation assays of specific genes via bisulfite sequencing PCR (BSP). DNA was first treated with bisulfate to convert unmethylated cytosines to uracils through deamination while methylated cytosines remained unchanged during the treatment. Uracils were converted to thymines during PCR. PCR primers were designed using Methyl Primer Express v1.0 (Table 2). PCR products were cloned and sequenced to determine the DNA methylation levels of specific genes using BiQ Analyzer software.

**Table 2:** Primers used for gene methylation studies.

Primer name^1^	Sequence (5′-3′)
ACSL1-f	TAGGTATTGTGGTGAAATYGTA
ACSL1-r	CCTAAATTTCACAAAAAATCC
FASN-f	GTGGTTTTAGGAGATAGTAAGGGT
FASN-r	TAACTTAAACAAAAAAATCTCCCT
SCD-f	GTTGGTATGGGTATAGGGATA
SCD-r	CAACCCAAAACTAACCCC
S6K1-f	TAAGGAAATYGAGGTTTTGA
S6K1-r	AAAAAAAAAACCACAACAAATCT
STAT5A-f	TTTAGGGTTTGAAATATTTGATT
STAT5A-r	TCAATTTTCTCATCTATCAAAAA

1 ACSL1, acyl-CoA synthetase long-chain family member 1; FASN, fatty acid synthase; SCD, stearoyl-coenzyme A desaturase; S6K1, ribosomal protein S6 kinase 1; STAT5A, signal transducer and activator of transcription 5A.

### Statistical analyses

The general linear model (GLM) of SPSS (v.18) was used in the data analysis. The GLM included the random cow effect and the fixed effect of diets, and mean differences for all variables were separated and compared using Duncan's multiple comparison procedure. Data are presented as means ± standard deviation. Significance was declared at *p*<0.05. Statistical correlation was performed using GraphPad PRISM 5.0, and standard error, *p*-value, and R^2^ were computed and used to evaluate the goodness of fit.

## Results

**Figure 1:**
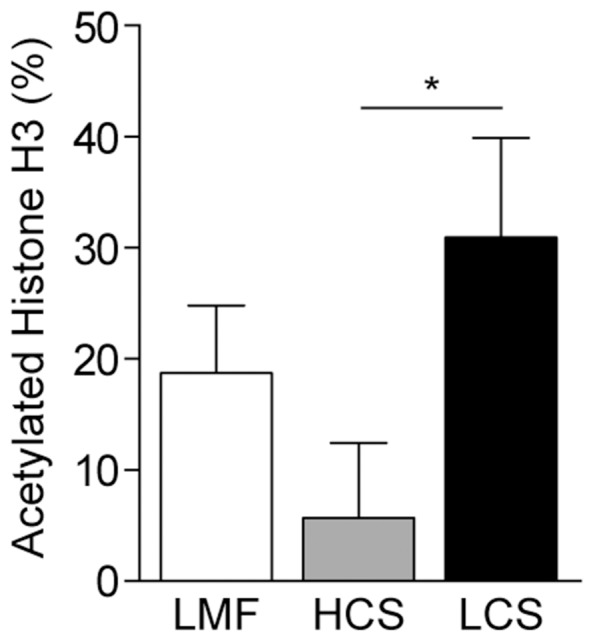
Effect of different diets on histone H3 acetylation levels in the mammary gland of dairy cows. The extent of histone H3 acetylation in the mammary glands of dairy cows was quantified using the EpiSeeker Histone H3 Acetylation Assay Kit. Data represent the mean and standard deviation (n = 10/group) and the asterisk indicates statistical difference (*p<0.05*) between the indicated columns. LMF, low-concentrate mixed forage diet; HCS, high-concentrate corn straw diet; LCS, low-concentrate corn straw diet.

We were interested in determining the impact of feeding the HCS diet on the epigenetic changes in the mammary tissue of dairy cows in comparison with the LCS diet and the LMF diet. We first analyzed the effect of these diets on the amount of histone H3 acetylation in mammary tissues. Extraction of histones and quantification of histone H3 acetylation revealed that acetylation was significantly reduced in cows fed the HCS diet, as compared with cows fed the LCS diet (*figure 1*). These data suggest that the high-concentrate diet may induce epigenetic changes in the mammary tissues.

**Figure 2:**
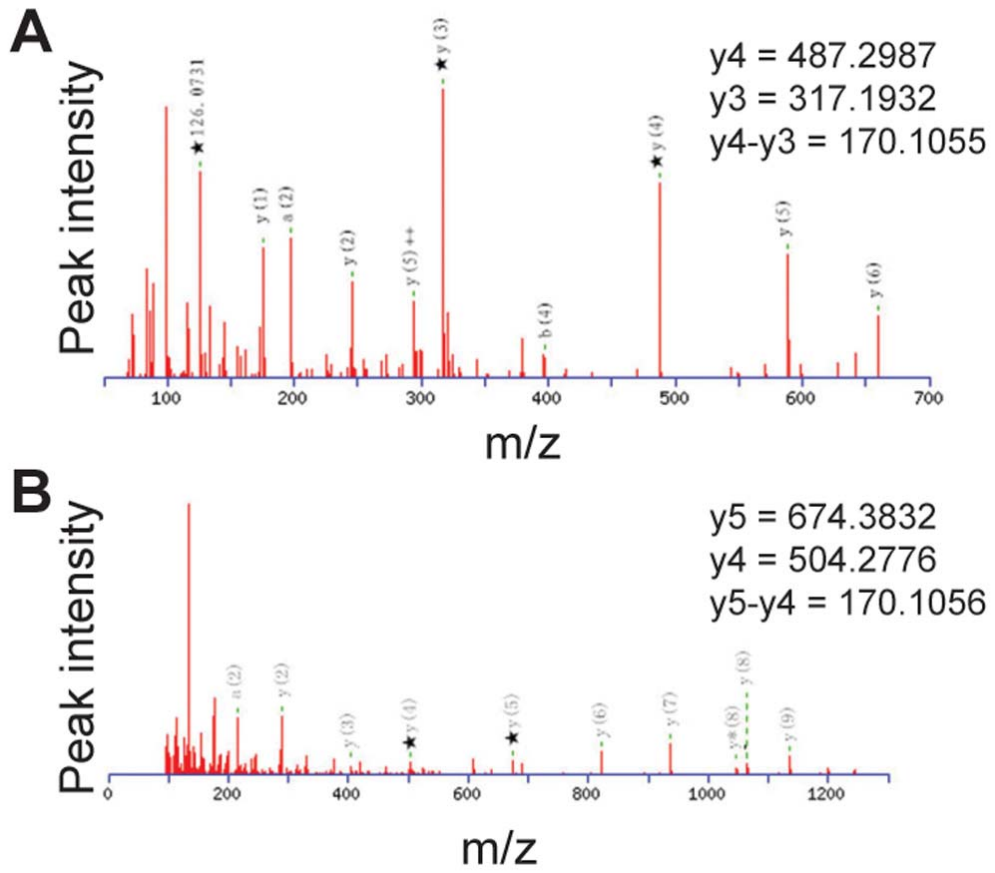
Mass spectra of histone H3 lysine-acetylated peptides identified using mass spectrometry. **A.** MS/MS spectra from m/z ion 882.4923. **B.** MS/MS spectra from m/z ion 1376.6936.

To validate these data, we further examined histone H3 acetylation by identifying specific acetylated peptides using mass spectrometry. Specific peaks diagnostic of lysine acetylation were identified (*figure 2*). Histone H3 acetylation occurred on lysines 5 and 7 (Table 3).

**Table 3:** Histone H3 acetylation sites identified in the mammary tissue of dairy cows.

Diet^1^	Molecular weight	Peptide site	Peptide sequence^2^	Acetylated site^3^
LMF	882.4923	20-27	K.QLATKA*AR.K	K5
	882.4923	20-27	K.QLATKA*AR.K	K5
	1376.6936	74-84	R.EIAQDFKT*DLR.F	K7
HCS	882.4923	20-27	K.QLATKA*AR.K	K5
	1376.6936	74-84	R.EIAQDFKT*DLR.F	K7
LCS	882.4923	20-27	K.QLATKA*AR.K	K5
	882.4923	20-27	K.QLATKA*AR.K	K5
	899.5188	20-27	K.QLATKA*AR.K	K5
	1376.6936	74-84	R.EIAQDFKT*DLR.F	K7

1 LMF, low-concentrate mixed forage diet; HCS, high-concentrate corn straw diet; LCS, low-concentrate corn straw diet.

2 The asterisk denotes the acetylated site.

3 K represents lysine.

**Figure 3:**
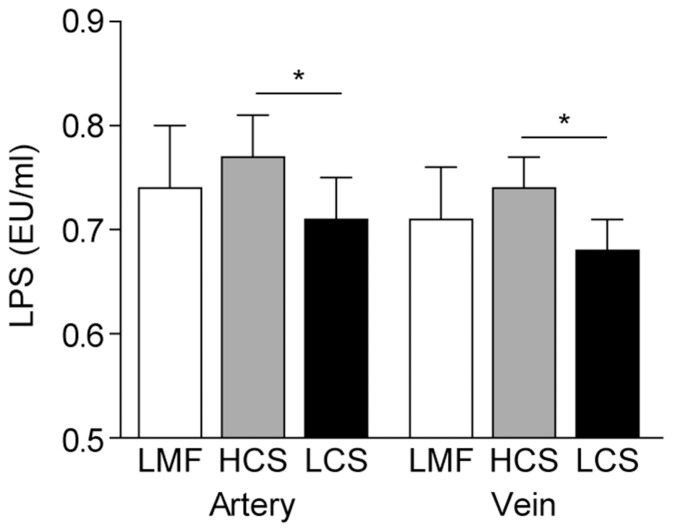
Effect of different diets on LPS concentrations in the mammary artery and vein plasma of dairy cows. LPS concentrations were quantified by chromogenic end-point assay using the limulus amebocyte lysate (LAL) test reagent kit. Data represent the mean and standard deviation (n = 10/group) and the asterisk indicates statistical difference (*p<0.05*) between the indicated columns. LMF, low-concentrate mixed forage diet; HCS, high-concentrate corn straw diet; LCS, low-concentrate corn straw diet.

A hallmark of feeding high-concentrate diets to cattle is the development of SARA [8] [16] [17], often resulting in the accumulation of LPS in the bloodstream, with subsequent inflammation [15]. To determine if the different diets affected LPS release, we quantified LPS in mammary artery and vein plasma samples. Cows fed the HCS diet had significantly higher LPS concentrations in the blood, as compared with cows fed the LCS diet (*figure 3*).

**Figure 4:**
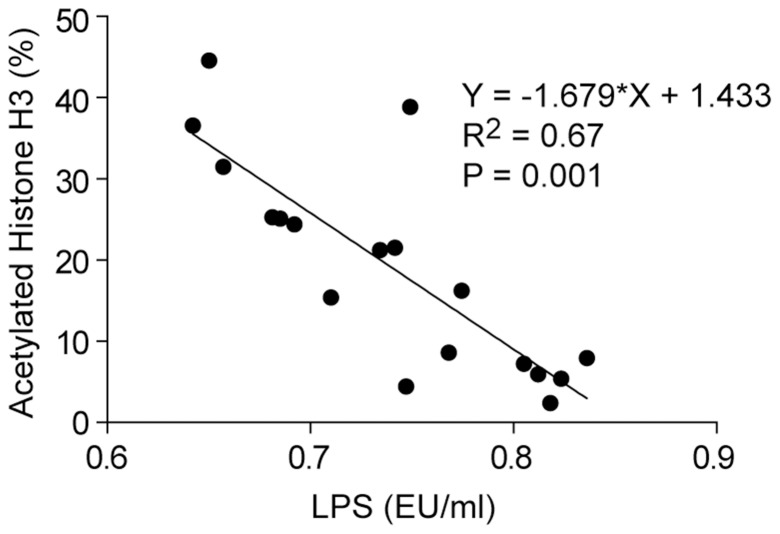
Relationship between mammary artery plasma LPS contents and histone H3 acetylation levels. The extent of histone H3 acetylation in mammary tissue samples was plotted vs. the LPS concentrations of mammary artery plasma samples.

We next determined whether LPS concentrations in the mammary blood were correlated with differing levels of histone H3 acetylation in the mammary tissue. We found that the extent of histone H3 acetylation was negatively correlated with LPS concentrations (*figure 4*).

**Figure 5:**
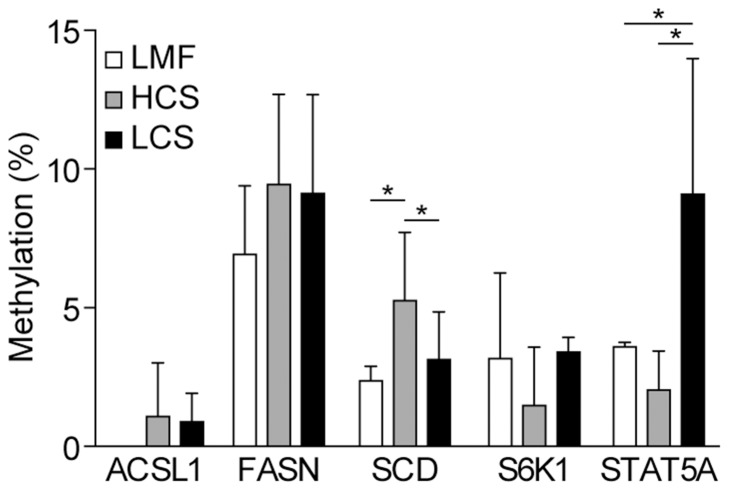
Effects of different diets on gene methylation levels in the mammary tissue of dairy cows. Gene methylation was analyzed using bisulfite sequencing PCR. Data represent the mean and standard deviation (n = 10/group) and the asterisk indicates statistical difference (*p<0.05*) between the indicated columns. ACSL1, acyl-CoA synthetase long-chain family member 1; FASN, fatty acid synthase; SCD, stearoyl-coenzyme A desaturase; S6K1, ribosomal protein S6 kinase 1; STAT5A, signal transducer and activator of transcription 5A. LMF, low-concentrate mixed forage diet; HCS, high-concentrate corn straw diet; LCS, low-concentrate corn straw diet.

The extent of DNA methylation of specific genes involved in fat and protein metabolism in the mammary gland was assessed using BSP. The methylation of the SCD gene encoding a stearoyl-coenzyme A desaturase associated with milk fat synthesis was increased in cows fed the HCS diet, as compared with cows fed either the LCS or LMF diets (*figure 5*). By contrast, methylation of STAT5A, encoding the signal transducer and activator of transcription 5A involved in protein synthesis, was reduced in cows fed the HCS diet (*figure 5*).

## Discussion

Histone H3 acetylation regulates various cellular physiological processes, including transcription, chromatin assembly, DNA replication and repair, and cell proliferation. Histone acetylation is associated with an “open” chromatin conformation that facilitates transcription [23] [24] [25]. The acetylation of histones can induce chromatin to adopt a more relaxed structure, thereby modulating the accessibility of DNA [26] [27]. We found that feeding cows the HCS diet decreased histone H3 acetylation as compared to feeding either the LCS diet or the LMF diet. In an earlier study of ours [28], we found that cows fed the HCS diet had identical dietary nutritional levels as compared with cows fed the LMF diet, but that various milk production parameters were negatively impacted by the HCS diet. Feeding HCS diets may affect milk gene expression so as to affect the milk component synthesis.

Our study showed feeding the HCS diet increased LPS concentrations in both mammary artery and vein plasma compared with cows fed the LCS diet. We also found that histone H3 acetylation levels were negatively correlated with LPS concentrations in mammary blood. It was shown previously that the LPS concentrations in both ruminal contents and blood increased after 21% dry matter in the control diet with a concentrate to roughage ratio of 50∶50 was replaced with a grain mixture comprised of 50% ground wheat and 50% ground barley [15]. The presence of LPS in the blood of dairy cows and goats fed diets containing a high proportion of concentrate was also reported in several studies [29] [30] [31]. Therefore, the lower histone H3 acetylation in the mammary tissue of cows fed the HCS diet can be attributed to the increased entry of LPS into the mammary tissue. Since there are few reports about the relationship of LPS and histone H3 acetylation in the mammary tissue of dairy cows to date, further studies are warranted to confirm the LPS-mediated histone hypoacetylation in the mammary tissue of dairy cows. Also unknown is the relationship between histone H3 acetylation and the physiological function of the mammary tissue in dairy cows.

DNA methylation is the oldest epigenetic mechanism that is known to correlate with gene repression [32]. The methylation status of DNA in promoter regions of specific genes efficiently regulates the transcription of the corresponding gene. High methylation tends to decrease the access of specific transcriptional factors to the promoter region of genes, whereas low or absent methylation results in an increased accessibility and transcriptional activity [33] [34]. For instance, a lower level of DNA methylation, measured as a percentage of 5′ methyldeoxycytidine, in the mammary tissue of Holstein cows was correlated with an increase in β-casein mRNA [35]. We found that feeding the HCS diet increased the methylation of genes (acyl-CoA synthetase long-chain family member 1, ACSL1; fatty acid synthase, FASN; and stearoyl-coenzyme A desaturase, SCD) associated with milk fat synthesis but decreased the methylation of genes (ribosomal protein S6 kinase 1, S6K1; and signal transducer and activator of transcription 5A, STAT5A) involved in protein synthesis as compared with feeding the LCS and LMF diets. We speculate that feeding the HCS diet could reduce milk fat synthesis but enhance protein synthesis in the udder. In support of this idea, the milk fat content for cows fed the HCS in this study was 3.58%, whereas the milk fat contents of cows fed the LCS and LMF diets were 3.95 and 4.23%, respectively. It has also been shown that when dairy cows were fed diets containing different proportions of barley grain, milk fat content was negatively correlated with barley content [14]. Several studies also demonstrated that both milk fat percentage and milk fat yield decreased during grain-induced SARA [15] [36] [37]. By contrast, SARA induction increases milk protein content [15] [38]. However, this increase in milk protein content may not necessarily reflect the rise of casein concentrations in milk, and in fact, casein content decreased after intramammary challenge with LPS [39]. The increase in milk protein is instead thought to be due to the rise of immune proteins such as acute phase proteins, albumin, β-lactoglobulin, lactoferrin, and antimicrobial peptides [40] [41] [42] [43].

Collectively, feeding a high-concentrate corn straw diet decreased histone H3 acetylation in the mammary tissue of dairy cows, primarily on K5 residues. Histone H3 acetylation levels were negatively correlated with LPS contents in the mammary plasma. LPS may play an epigenetic regulatory role in the mammary tissue of dairy cows. Feeding a high-concentrate corn straw diet tended to increase the methylation of genes associated with fat synthesis but decrease the methylation of genes associated with protein synthesis.
